# Voltammetry of Carbon Nanotubes and the Limitations of Particle-Modified Electrodes: Are Carbon Nanotubes Electrocatalytic?

**DOI:** 10.1021/acs.jpclett.2c02464

**Published:** 2022-09-12

**Authors:** Archana Kaliyaraj Selva Kumar, Yuanyuan Lu, Richard G. Compton

**Affiliations:** Department of Chemistry, Physical and Theoretical Chemistry Laboratory, Oxford University, South Parks Road, Oxford OX1 3QZ, Great Britain

## Abstract

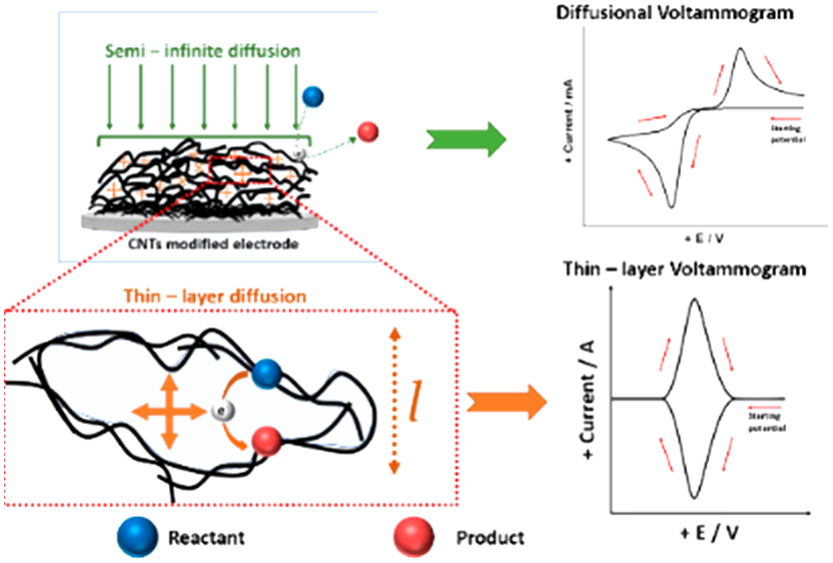

The use of carbon nanotubes (CNTs) as electrocatalysts is summarized; the limitations of using voltammetry based on CNT-modified electrodes is explained; and the role of mass transport, as well as electrode kinetics, with respect to dictating the voltammetric responses is discussed. The use of single-entity electrochemistry to at least complement, if not replace, ensemble voltammetry is advocated along with other caveats, notably purity, with respect to CNT voltammetry.

Carbon nanotubes (CNTs) emerged as a novel nanomaterial following their discovery in 1991 by Ijima^1,2^ and, as is well-known, have subsequently received vast attention across a plethora of areas.^3−5^ The familiar structure of CNTs takes the form of “rolled-up” graphite sheets^6^ with two principal types, namely, single-walled carbon nanotubes (SWCNTs) and multiwalled carbon nanotubes (MWCNTs). The diameter of the CNTs ranges from a few to tens of nanometers, and the lengths are generally of the order of micrometers. The morphology of the MWCNTs depends on the synthetic route by which they are made. The two most important routes are chemical vapor deposition or the high-voltage discharge arc method. Thus, MWCNTs can be formed as “hollow-tubes”, “herringbone”, or “bamboo-like” structures.^6^ By analogy with graphite, the ends of the CNTs can be described as “edge-plane-like” and the walls as “basal-plane-like”. Hence, both “herringbone” and “bamboo-like” MWCNTs have huge numbers of “edge-plane-like” sites that are thought to be responsible for some of their electrochemical properties.^7−11^

CNTs find wide application for electrochemical sensing and electrocatalysis often in the form of modified electrodes. CNT composites are widely used as electrodes and/or substrates for energy conversion and storage. A few typical examples where CNTs have been used effectively for electroanalysis are given in Table S1 in the Supporting Information, and the applications in energy conversion are illustrated by the selected examples given in Table S2.

From Tables S1 and S2 it can be inferred that CNT ensembles play a major role in electrocatalysis underpinning many different applications in sensors, fuel cells, and batteries. Electrodes modified with CNTs are usually aimed at promoting the electron transfer reactions of interest to the particular application. The faster, or otherwise, electron transfer kinetics of these modified electrodes is invariably analyzed using cyclic voltammetry.^12^ The criterion used is based on a reduced peak-to-peak separation of the redox peaks in a cyclic voltammogram as shown schematically in Figure 1 typically relative to the unmodified electrode. Figure 2 shows authentic examples of the many in the literature of voltammograms comparing CNT-modified electrodes to bare GC electrodes. The origin of this diagnostic has a basis in the well-developed theory of irreversible electron transfer kinetics within the Butler–Volmer^13−15^ or the Hush–Marcus^16−18^ frameworks at a flat, planar electrode. The extent to which this is applicable to modified electrodes is critically discussed in the following section which summarizes the origin of the relationship between the peak-to-peak separation and the rate of electron transfer.

**Figure 1:**
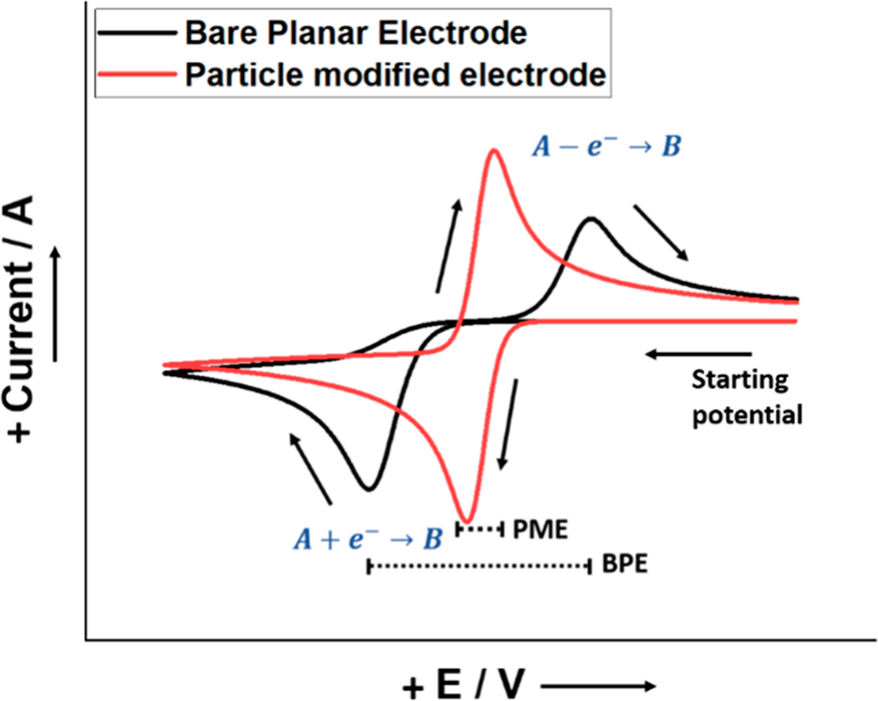
Representative cyclic voltammograms at a bare planar electrode (BPE) and at a particle-modified electrode (PME) for the one-electron reduction of A to B showing a reduced peak-to-peak separation for a PME compared to BPE electrode which is often thought to indicate “electrocatalysis”. Both A and B are solution-phase species. The dotted line shows the peak-to-peak separation at the corresponding working electrode.

**Figure 2:**
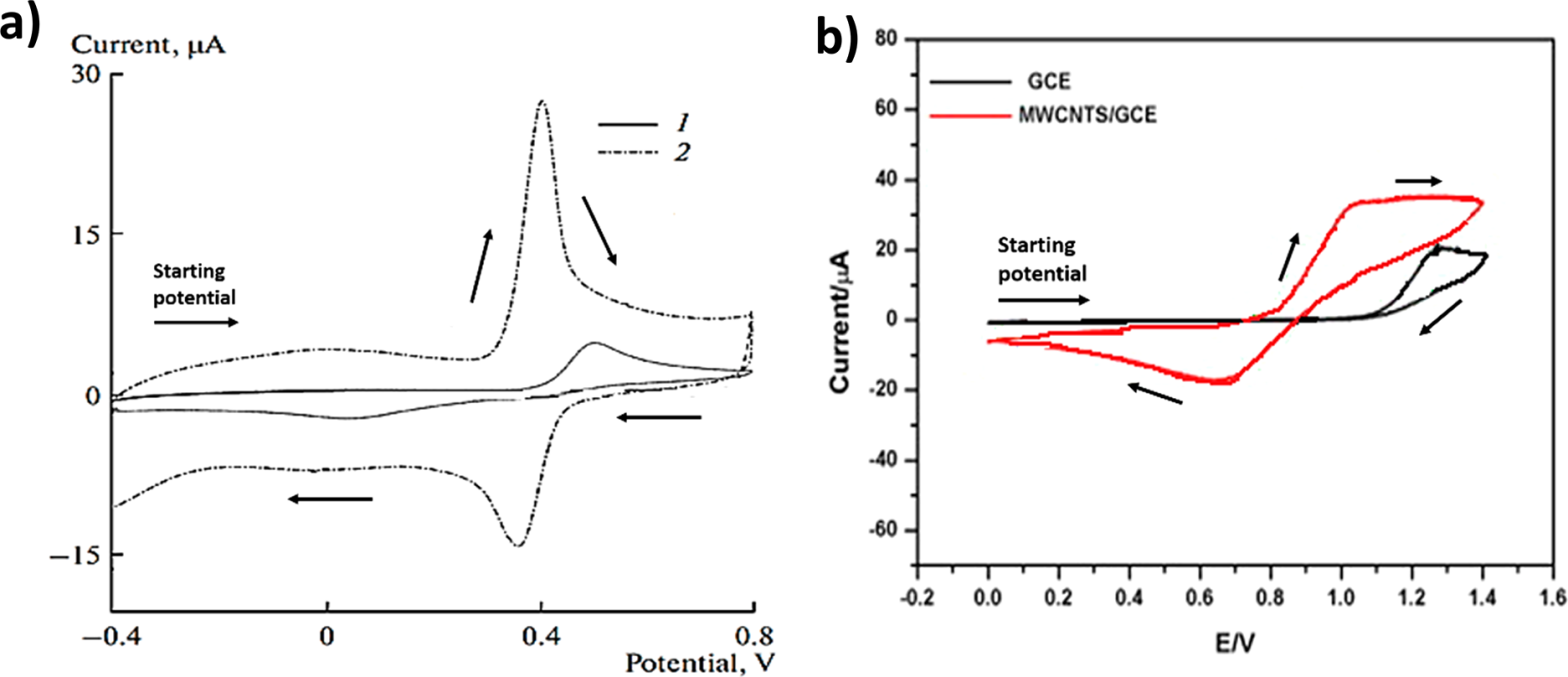
(a) Cyclic voltammograms at a bare glassy carbon electrode (GCE) (1) and a MWCNT-modified GCE (2) in 1 × 10^–3^ M paracetamol at a scan rate of 100 mV s^–1^. Supporting electrolyte: 0.1 M phosphate buffer solution with pH 6.5. Reproduced with permission from ref (19). Copyright 2022 Springer Nature. (b) Cyclic voltammograms of the GCE and MWCNTs/GCE, at scan rate of 10 mV s^–1^ in 0.1 M VOSO_4_ + 2 M H_2_SO_4_. This figure is adapted with permission from ref (20). Copyright 2011 Elsevier.

We next consider cyclic voltammetry at flat, planar electrodes. According to Butler–Volmer kinetics the rate of interfacial electron transfer is determined only partly by the standard electrochemical rate constant *k*^0^, the electrode potential, and the transfer coefficients. Importantly it also reflects the concentration of the redox couple local to the electrode surface. Hence, the voltammetry observed for the reaction is determined by the magnitude of the electrochemical rate constant and also the prevailing rate of mass transport to the electrode. The electrocatalytic activity of an electrode surface is reflected in the former but not the latter. Electrolysis leads to the depletion of the electroactive reactant local to the electrodes leading to a zone of depletion (“diffusion layer”) adjacent to the electrode. The rate of mass transport can be crudely estimated using the mass transport coefficient *m*_T_
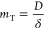
1where δ is the diffusion layer thickness at a planar electrode undergoing a voltammetric scan and which depends approximately on the duration of the electrolysis time *t* by
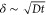
2where, in the case of a linear voltage sweep, *t* is the time taken to scan the voltammogram across the given potential and is given by
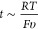
3where υ is the scan rate in *V* per second. Equation 2 assumes semi-infinite linear diffusion of the analyte to the planar electrode surface as shown in Figure 3.

**Figure 3:**
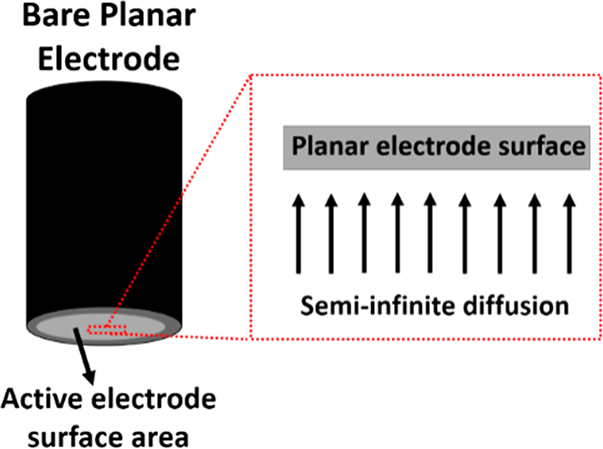
Schematic representation of semi-infinite diffusion at planar macroelectrodes.

In such cases, the electrochemically active electrode area is uniformly accessible with the contributions from the electrode edge negligible since the size (radius) of the electrode is much greater than the diffusion layer thickness.^21^ The term “semi-infinite” implies that the expansion of the diffusion layer into the solution does not meet any boundaries or obstacles. Equations 2 and 3 combined with Fick’s first law of diffusion imply that the voltammetric peak current under semi-infinite diffusion conditions scales linearly with the square root of scan rate.

Equation 1 can be rewritten as
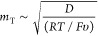
4The relative magnitudes of *m*_T_ and *k*^0^ dictate the observed voltammetry, including the peak-to-peak separation. In the so-called electrochemically reversible limit
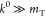
5while for an electrochemically irreversible reaction
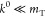
6The quantitative criteria for the transition between the electrochemically reversible and irreversible limits were developed quantitatively by Matsuda and Ayabe.^22^ Note that electrochemical reversibility relates to electron transfer and not to the chemical stability or otherwise of the species A and B. It is possible for a redox process to be electrochemically reversible (fast electron transfer compared to diffusion) but chemically irreversible (where the species B decays on the voltammetric time scale so no back peak is seen on the reverse voltammetric scan).

Figure 4 shows representative cyclic voltammograms for electrochemically reversible, quasi-reversible, and irreversible process (with full chemical reversibility). Here, for reversible voltammogram the separation between oxidative and reductive peak is less when compared to the quasi-reversible voltammogram and much less than the irreversible voltammograms. In addition, from the fully irreversible to the fully reversible process there is a small increase in the peak current on the forward scan, but as *k*^0^ increases the peak current reaches a maximum value controlled by the largest diffusional flux consistent with the electrode potential, while as *k*^0^ decreases the peak current decreases to a finite minimum limiting value. In this “irreversible limit”, as *k*^0^ decreases further the voltammetric wave shifts to more negative potentials (as an “overpotential” needs to be applied to overcome the kinetic barrier) but the peak current remains unchanged. The difference in peak currents in the two limits for a one-electron transfer at the same voltage scan rate is just a factor of ca. 30%,^23^ which is a remarkably small difference. The effects of electrocatalysis overwhelmingly change the peak potential not the magnitude of the peak current.

**Figure 4:**
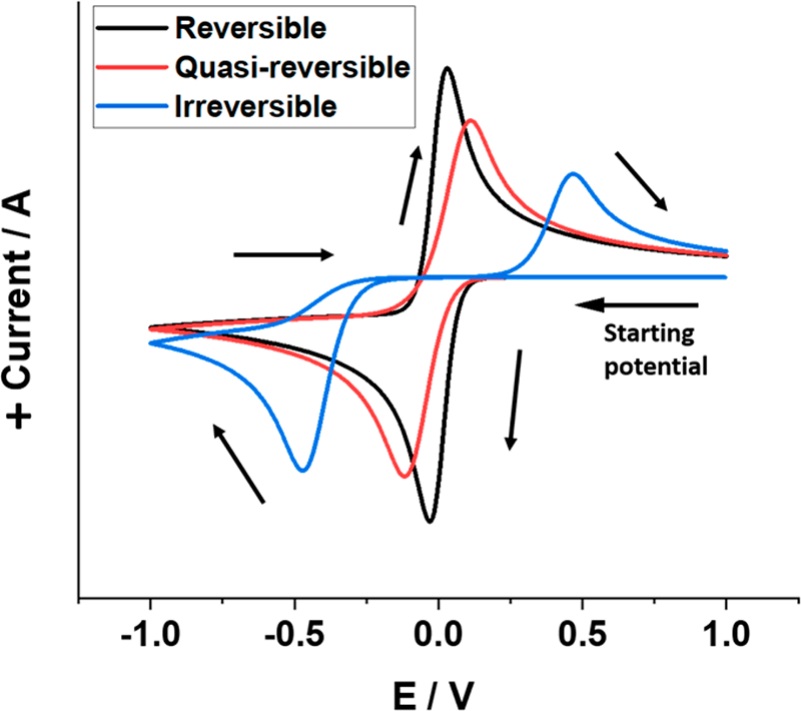
Schematic representation of reversible, quasi-reversible, and irreversible cyclic voltammograms for the single electrode transfer A(aq) + e^–^ → B(aq).

It follows that the extent of the electrochemical reversibility, and so the extent of electrocatalysis, as quantified by the magnitude of *k*^0^ is reflected in the peak-to-peak separation, *ΔE*_pp_, of the cyclic voltammograms is given by
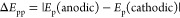
7where *E*_p_(anodic) and *E*_p_(cathodic) are the peak potentials of the oxidative and reductive peaks, respectively.

*ΔE*_pp_ changes at low scan rates, from the reversible limit where, for a one-electron process
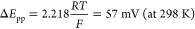
8to high scan rates, the irreversible limit where, again for a one-electron process
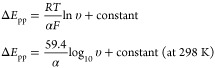
9Hence, in the reversible limit *ΔE*_pp_ is approximately 57 mV (at 298 K) and is independent of the scan rate, whereas under quasi- and irreversible conditions *ΔE*_pp_ depends on the scan rate and the electrochemical rate constant. The peak-to-peak separation increases with scan rate but decreases with an increase in the rate constant. Thus, in comparing different redox couples at a particular electrode or the voltammetric response of a particular A/B couple at different surfaces it is reasonable to use the peak-to-peak separation as a qualitative guide to the relative extent of electrocatalysis. These conclusions however assume semi-infinite diffusion. In the next section we discuss voltammetry under thin-layer conditions where the expansion of the diffusion layer during electrolysis is limited by the close proximity of a boundary to the electrode and thus serves to spatially confine the diffusion layer.

We now consider thin-layer voltammetry. Electrolysis of species in solution phase occurs under “thin-layer” conditions when the diffusion layer thickness  reaches the boundary of the electrochemical cell early in the voltammetric scan. In such cases the current response of an electrochemically reversible process is more symmetrical than seen for semi-infinite diffusion as shown in Figure 5. Note the absence of the “diffusional tail” seen for semi-infinite diffusion where the current decays 1/√*t* at potentials and times beyond the peak current.

**Figure 5:**
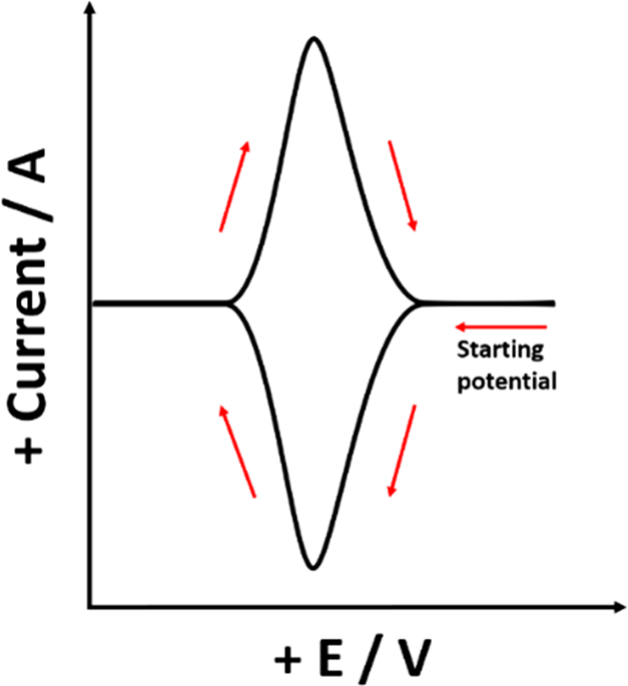
Representative cyclic voltammogram under thin-layer conditions showing symmetric redox peaks for the electrochemically reversible process, A(aq) + e^–^ → B(aq).

For a reversible one-electron transfer reaction (A + e^–^ → B) under thin-layer conditions the peak potential is similar to that of the formal potential *E*_p_ = *E*_F_^O^ and the peak current is given by
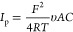
10where υ is the scan rate; *A* is the area of the electrode; and *C* is the concentration of analyte, A, assumed to be uniform in the thin layer prior to electrolysis when the initial concentration of B is zero. Note that in the limit of full thin-layer behavior the concentrations of A and B remain uniform throughout the layer at all times at levels controlled by the electrode potential^24^ as shown in Figure 6 where the contrast with the concentration distributions under semi-infinite diffusion is important to note. The peak-to-peak separation is independent of the scan rate and equal to zero since diffusional hysteresis is absent. The peak potentials correspond to the formal potential of the A/B couple.

**Figure 6:**
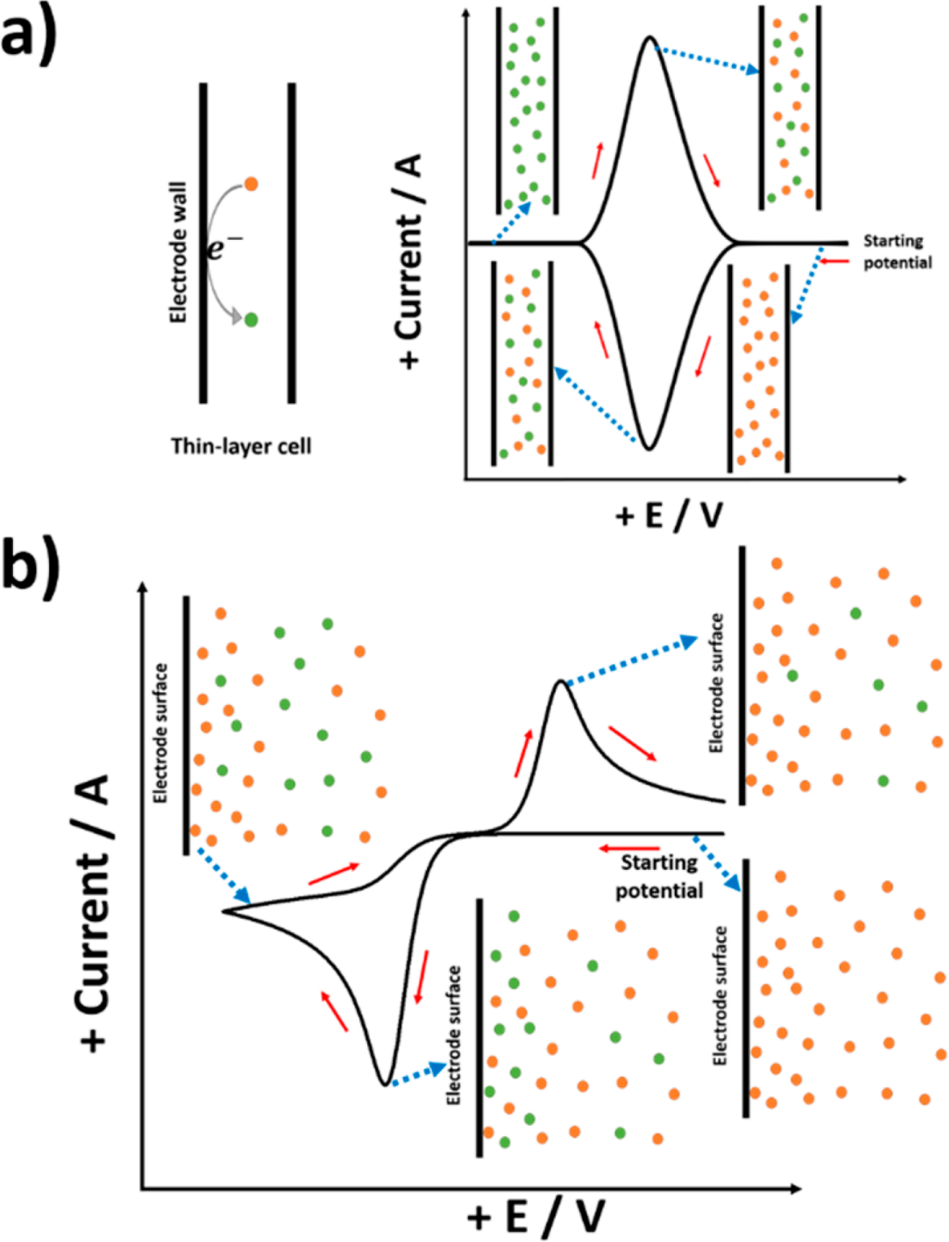
Schematic diagram showing the reactant (orange) and product (green) concentrations in (a) a thin-layer cell and (b) under semi-infinite diffusion for an electrochemically irreversible one reduction at flat, planar electrode surface.

If the same solution-based redox couple is compared under thin-layer or semi-infinite diffusion conditions with all other parameters remaining the same, then the volume of solution electrolyzed during the voltammetric scan is much smaller under thin-layer conditions since the corresponding diffusion layer is constrained. The consequence is that voltammetric peaks which appear irreversible under semi-infinite diffusion conditions appear more reversible under thin-layer conditions. Thus, the peak appears either at, or closer to, the formal potential of the A/B couple under thin-layer conditions than under conditions of semi-infinite diffusion.

Note that the total charge under the *i*–*E* curve in Figure 5 is independent of υ as this corresponds to the complete electrolysis of all the analyte in the thin-layer cell.

In general, if measurements of the peak potential are made as a function of scan rate the diffusional character ranging between the extreme limits of semi-infinite and thin-layer diffusion can be quantified by means of a log–log plot of the peak current against scan rate, the slope of which will lie between 0.5 and 1.0 corresponding to the two limits with intermediate values indicating a mixed character. Such plots are useful in the study of CNT-modified electrodes which are next considered.

We next introduce diffusion at CNT-modified electrodes. The electrochemical behavior of CNTs is usually studied by drop-casting them on a substrate electrode.^25−27^ A schematic representation of the drop-cast technique is given in Figure S1 in the Supporting Information. In this method, the CNTs are suspended in a solvent and a known volume of the aliquot is dropped onto the substrate electrode; the solvent is removed via evaporation before use. Such an electrode is denoted as a CNT-modified electrode (CME).

Table S3 gives representative examples taken from the literature of inferred “faster electrode kinetics” for diverse redox reactions at a CME than at the substrate electrode, often glassy carbon, and so is generically interpreted as “electrocatalysis” by the nanotubes. This conclusion is drawn voltammetrically from observing a decreased peak-to-peak separation at the CME than at the substrate electrode. However, such comparison is valid only if both the electrodes have similar mass transport regimes since the peak potential reflects a point of balance between the electrode kinetics and the rate of mass transport; the reduction may reflect a change in either or both of these quantities.

Usually a CME is made with a thick layer of CNTs so that the redox reaction of interest occurs fully at the CNTs rather than at the underlying substrate electrode. For this, a strong overlap of the diffusion layers of each CNT is required such that the coverage leads to overall linear diffusion to the CME. According to Einstein’s theory, for a voltammetric time scale *t*, the CNTs need to be within an approximate distance of δ = (6*Dt*)^1/2^ from one another for a strong overlap^28^ so that the particles dominate the signal and not the substrate electrode. For instance, when *D* = 10^–5^ cm^2^ s^–1^, a typical aqueous solution value, over a time scale of just a few seconds, δ can become as large as ca. 100 μm. Thus, for a random distribution of particles on a substrate electrode, a coverage of 10^5^ particles cm^–2^ is required to ensure diffusional overlap. Usually in experiments very much larger amounts of particles are deposited, as shown in Figure 7, on the substrate electrode.^29^

**Figure 7:**
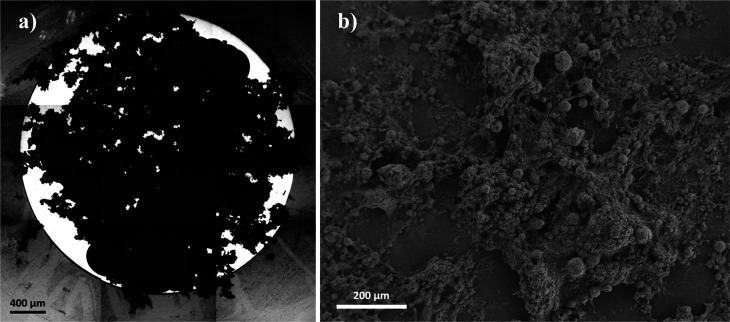
(a) Optical microscope images for a GC electrode with a geometric area 0.07 cm^2^ which was drop-casted with 30 μg of CNTs and (b) SEM images of CNTs drop-casted over a carbon slab.

For most CMEs we can assume that the voltammetric signal arises exclusively from the CNTs and not the substrate electrode. The drop-casted CNTs on a substrate electrode usually exist as a thick porous layer with many layers of CNTs and in which the nanotubes agglomerate/aggregate randomly and form pockets of solution as implicit in the SEM images in Figure 7. It can be inferred that during the electrolysis process the modified electrode shows semi-infinite diffusion to the surface of the electrode. However, inside the porous structure the electrolyte is trapped in CNT-bounded pockets. Given that the average CNT-to-CNT separation is small compared to the distance diffused on the voltammetric time scale and that the CNTs are conductive, this resembles a thin-layer cell as discussed in the previous section.^30−32^ Electrolysis within the film thus takes place under thin-layer conditions. Under such circumstances, and as discussed above, the peak potential shifts closer to the formal potential of the redox species than the peak potential measured under semi-infinite diffusion condition all other factors remaining constant.^31^

In general (for both electrochemically reversible and irreversible reactions), voltammetry at a CME will show two signals for a single redox process. One signal arises from semi-infinite diffusion to the surface of the electrode mirroring that expected for an approximately flat nonporous surface with the electrochemical characteristics of CNT surfaces, while the other signal, observed nearer to the formal potential of the redox process of interest, that is to say with lower overpotential, is due to the electrolysis of reactant trapped in the porous structure and displays characteristics of “thin-layer” voltammetry.^33,34^Figure 8a shows a schematic representation of both semi-infinite diffusion to the CME surface and thin-layer electrolysis at the porous pockets. Figure 8b shows typical simulated voltammetry showing the splitting of peaks corresponding to the “thin-layer” and semi-infinite diffusion peaks. The arrows in Figure 8b show that as the amount of analyte in the porous layer increases the former peak increases relative to the latter peak. The simulated voltammogram is reconstructed from ref (33).

**Figure 8:**
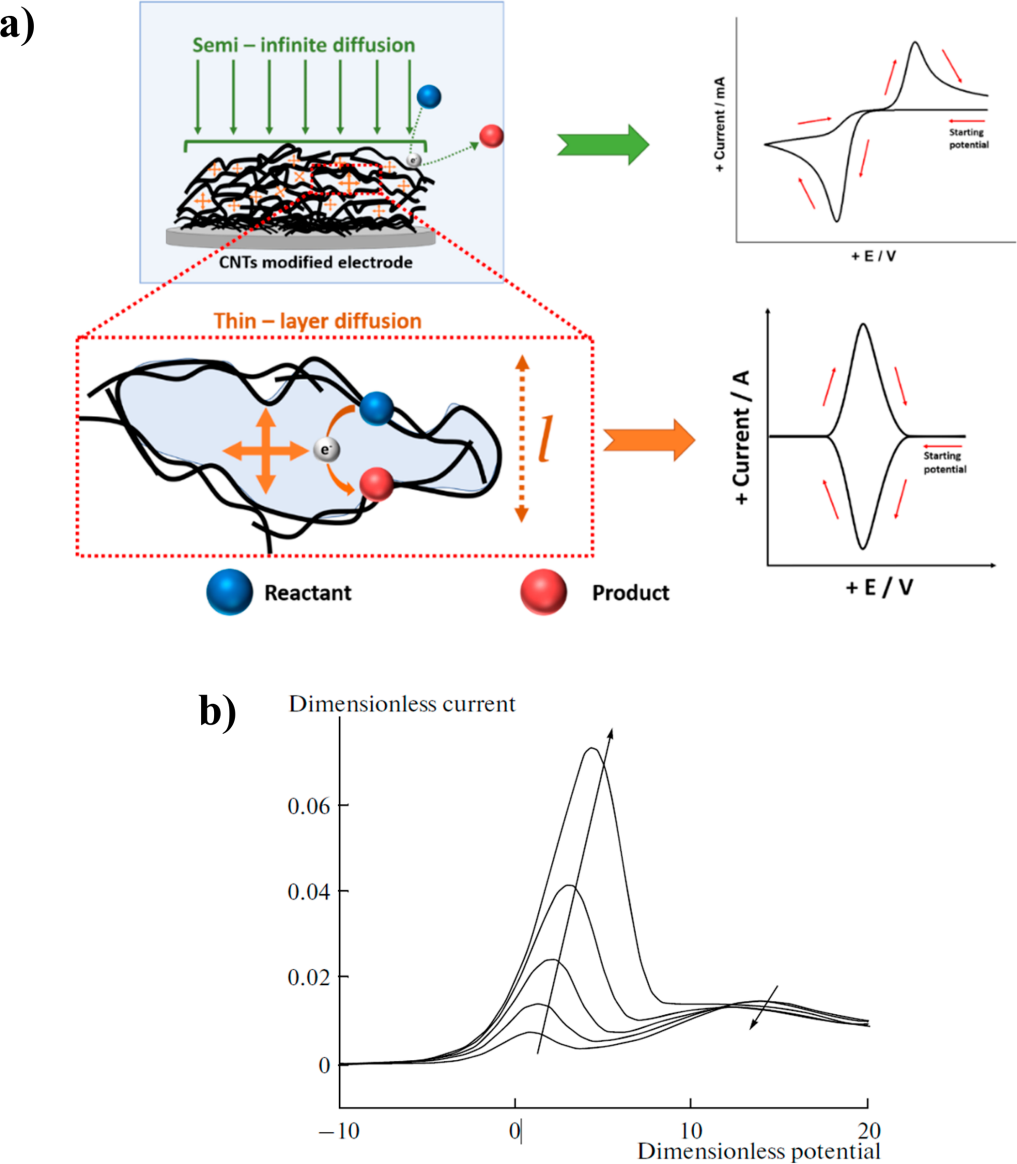
(a) Schematic representation of semi-infinite diffusion at the surface of CNTs (green arrows) and thin-layer diffusion (orange crossed arrows) in the porous pockets formed by CNTs along with their corresponding voltammetric signals and (b) simulated voltammetry showing the two peaks seen for a single redox process arising from both “thin-layer” and semi-infinite diffusion. The arrows show an increase in the peak current for the thin layer relative to that for semi-infinite diffusion as the thickness of the porous layer increases. The figure is reproduced with permission from ref (33). Copyright 2022 Springer Nature.

Note that the relative sizes of the two signals at a given voltage scan rate are controlled by the size of the porous layer of CNTs since the magnitude of the semi-infinite diffusion signal is essentially independent of this parameter while the charge passed in the thin-layer signal scales with the extent of the CNT layer. In extreme cases the latter dwarfs the former, which can appear negligible. However, the relative sizes of the two peaks are scan-rate-dependent with the thin-layer signal scaling approximately linearly with scan rate whereas the diffusional signal scales approximately with the square root of scan rate. The interplay of semi-infinite diffusion and thin-layer effects is generic to electrodes modified with nanomaterials; a recent report indicated the presence of two signals for the oxidation of hydrazine at electrodes modified with palladium nanoparticles.^35^

Given the complexity of the voltammetric response seen at CMEs it is interesting to briefly comment on the voltammetry observed when an electrode surface is partially “blocked” by layers of inert (nonadsorbing, nonconducting) particles which simply serve to change the rate of diffusion of a redox species to the electrode surface; the spheres themselves play no other role than to physically occupy space within the diffusion layer of the electrode so that electrolysis is exclusively constrained to take place at the electrode surface and not, as with CMEs, within the porous layer. Figure 9 schematically illustrates the partially blocked electrode.^36,37^

**Figure 9:**
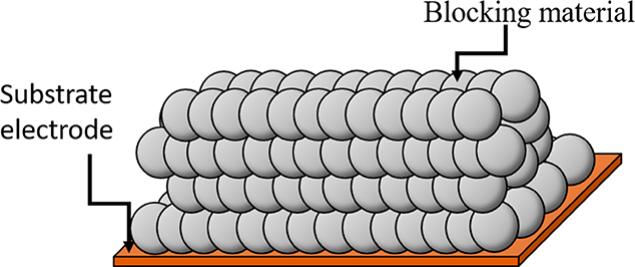
Schematic representation of substrate electrode partially blocked by nonconductive particles.

Even for a fully electrochemically reversible reaction, because of the interplay of semi-infinite diffusion and thin-layer constraints inside the porous layers formed by the nonconductive nanoparticles the voltammetric signal is reduced in magnitude, but importantly, the peak-to-peak separation can increase or decrease relative to what is seen at the bare electrode even when the process is constrained to be fully Nernstian (reversible) in simulations! Such results are, at first sight, intuitively interpreted as positive or negative catalysis. The exact responses depend on the voltage scan rate (in the case of linear sweep voltammetry), the number of monolayers of particles, and the particle size.^34,36−38^ That such complexity in the voltammetric response is apparent even for the simplest possible case—that of a fully reversible process at a partially blocked electrode covered in a regular layered array of identically sized spheres—cautions against the drawing of firm conclusions with respect to “electrocatalysis” using the cyclic voltammetry of CMEs or other particle-modified electrodes alone. Thus, to understand the intrinsic catalytic property of nanoparticles, including CNTs, the “nanoimpact” technique is adopted where single nanoparticles are studied as discussed in the next section.

Now, we consider the single-entity (aka “nano-impact”) approach to explore the possible electrocatalysis at a CNT. The single-entity approach has been developed to explore the electrochemistry of single nanoparticles and has been well reviewed.^39−41^ In brief, an electrode, usually a microelectrode, held under a controlled potential is inserted in a dilute suspension of the nanoparticles of interest. From time-to-time by virtue of their Brownian motion individual particles impact the electrode surface. This may lead to semipermanent sticking (irreversible adsorption) of the particle to the surface or, at the other extreme, the particle remains in solution but, for a short time, often milliseconds, close enough to the electrode to permit electron transfer to and from the particle. If the solution contains a redox couple, A/B, and if the impacting particle is more electrocatalytic toward the couple than is the microelectrode, then for conductive particles such as Ag or Pt nanoparticles or CNTs, the particles adopt the potential of the electrode for the duration of the “impact”, and the A/B redox reaction may be mediated via electron transfer from the electrode through the nanoparticle to the solution phase species. If the electrode potential, and hence that of the transiently impacted particle, is sufficient to drive the A/B redox chemistry then a current spike is observed for the duration of an impact the duration of which reflects the period of time the particle has electrical contact. For sufficiently long impacts the spike is seen as a step, as illustrated in Figure 10.

**Figure 10:**
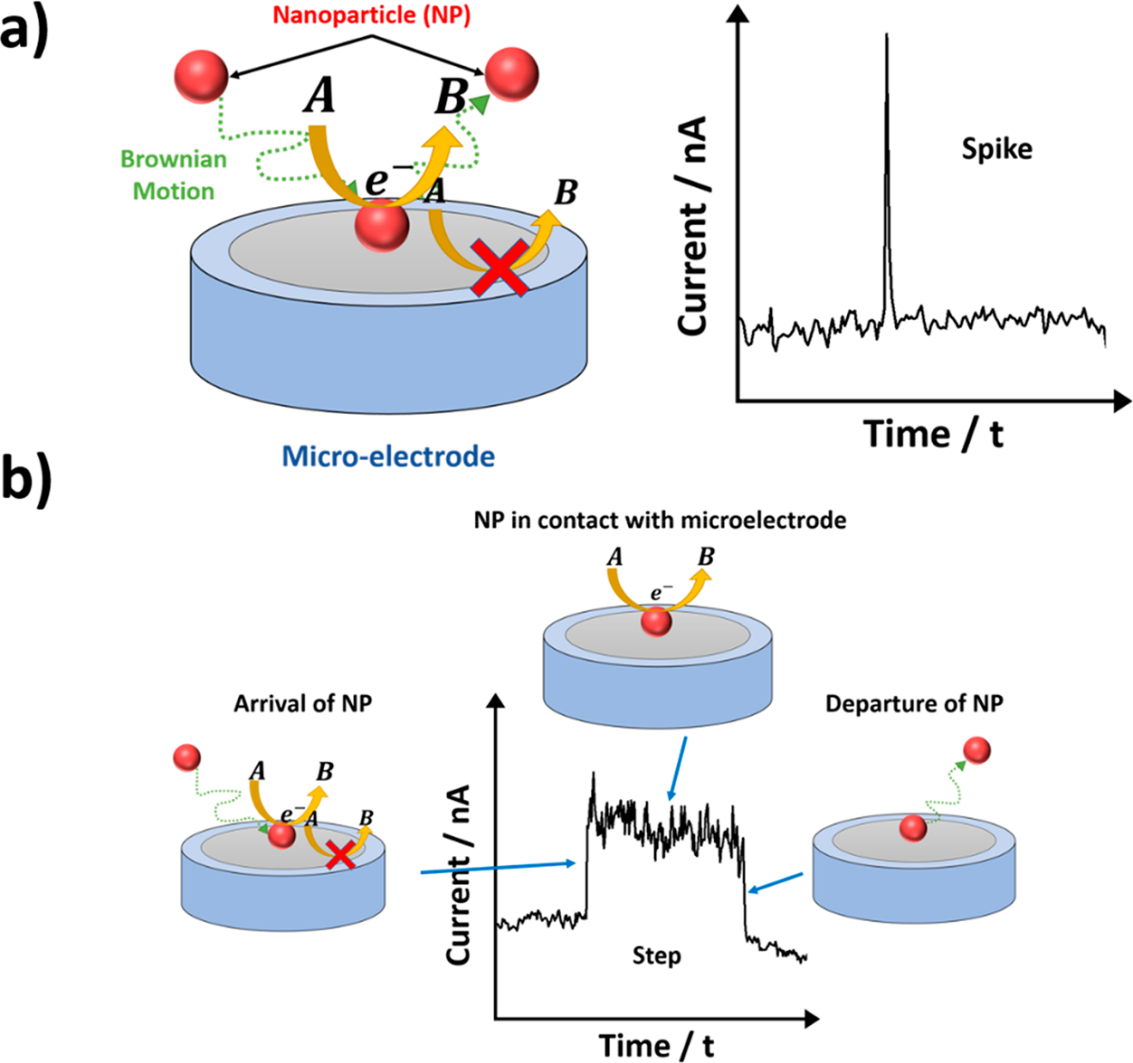
Schematic representation of (a) shorter duration impacts resulting in current spikes and (b) a longer residence time at a microelectrode showing current steps. The arrows in yellow denote the mediated one-electron reduction reaction A(aq) + e^–^ → B(aq).

The nanoimpact method has been used to study nanoparticle catalysis at metal nanoparticles, for example, the reduction of protons^42^ or the oxidation of hydrazine^43^ at Pt particles. In the case of silver nanoparticles, the application of a suitably oxidizing potential can lead to the dissolution of the particles as Ag^+^ cations,^44^ an observation which allows the detection of single viruses by tagging with Ag nanoparticles.^45^ In the case of CNTs decorated with tiny Pd nanoparticles, impacts at a carbon nanowire electrode in the presence of dilute acid were revealed by virtue of the Pd modification causing electrocatalysis of the underpotential reduction of protons to adsorbed H atoms. The charge passed during the impact event permitted an estimate of the length of the CNTs giving good values in comparison with independent measurements of CNT length via SEM. The decorated tubes were thus shown to be active along their entire length.^46^ The duration of the impact was seen to be of the order of tens or hundreds of seconds, which was ample to allow the measurements of both chronoamperograms (measurements of current vs time at a fixed potential) and cyclic voltammograms while the CNT was in electrical contact with the nanowire. In this way the quasi-steady-state currents flowing to the impact single CNTs^46^ were seen to reach a limiting value at high potentials consistent with the diffusion-controlled reduction of protons to hydrogen at the single CNT modeled as a cylinder while the single CNT cyclic voltammograms allowed inference of the electrode kinetics of proton reduction at the single (Pd-modified) CNT. The noise present on the steady-state currents was interpreted in terms of the modulation of the distance between the substrate electrode and the CNT caused by motion of the CNT, and the electrical resistance associated with the electrode-to-tube contact was estimated in an experiment in which a CNT impact was used to bridge between two closely spaced microband electrodes.^47^ The single-entity cyclic voltammetry method was used to investigate both the oxygen reduction and formate oxidation reactions at single Pd-decorated CNTs.^3,48^

Undoped single MWCNT voltammetry has been compared with that from drop-casted MWCNT ensembles with respect to the catalysis of the bromide/bromine redox couple of importance in diverse flow battery systems.^49^ While an *apparently* nearly fully electrochemically reversible response was seen for the latter, the single-entity measurements showed that the CNTs were only mildly catalytic in comparison with glassy carbon; the mass transport regime in the porous CNT films masked the true catalytic response. The conclusions about the extent of electrocatalysis were drawn by measuring the impact currents at the single CNTs as a function of potential for both bromide oxidation and bromine reduction as shown in Figure 11. The plots of current versus potential shown correspond to the quasi-steady-state voltammograms for the two electrode processes at single CNTs and, with a knowledge of the relevant formal potential for the bromine/bromide couple, can be analyzed to give a quantitative assessment of the extent of electrocatalysis. Similar comparative observations were made with respect to the VO_2_^+^/VO^2+^ couple except that the multistep reaction involving bond breaking/making and proton transfer steps in combination with electron transfer was shown to be catalyzed by CNTs in the oxidative direction but retarded in the reductive direction!^50^ The single-entity approach has also been used to reveal the role of surface oxygen functionality, such as, and especially quinones, in the reduction of oxygen in aqueous base.^51,52^ We note that beyond electrocatalysis the electro-oxidation of the CNTs themselves either undoped or with amino functionalization has been characterized.^53^

**Figure 11:**
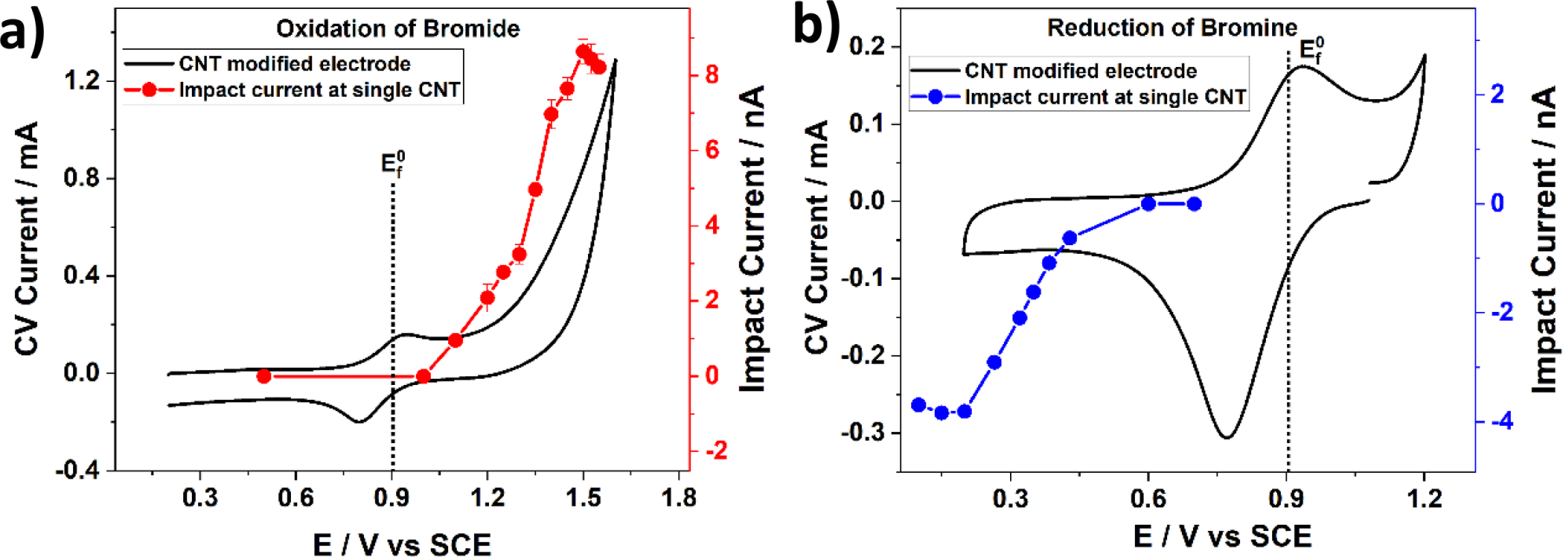
Single-entity CNT impact currents measured as a function of potential and the corresponding ensemble with cyclic voltammograms at CNT-modified electrodes for (a) the oxidation of bromide in 5 mM NaBr (red circles) and (b) the reduction of bromine in 5 mM bromine (green circles). The dotted lines in both graphs show the formal potential, *E*_f_^0^, of the Br_2_/Br^–^ redox couple.

In all the above applications of single-entity studies to reaction of or at CNTs, the investigation of the electrode processes takes place under much better-defined conditions of mass transport than occur at macroelectrodes modified with porous layers of many CNTs. In diverse studies, a simple mass transport model of diffusion to a cylinder has found quantitative success^3,46−48,54^ in contrast to the situation where the voltammetric signal has two variably sized components reflecting both semi-infinite diffusion to the layer surface and thin-layer type diffusion within a porous layer in combination with uncertain coupling between the two. A recent study^55^ highlighted the relative merits with respect to clarifying voltammetry relating to species which might adsorb on CNTs. In the case of a CNT-modified electrode, particularly using a thick coating of CNTs, the large voltammetric signals attributed to the species within the coating scales linearly with the voltage scan rate, consistent with electrolysis under thin-layer conditions. However, such a response would also be seen if the species adsorbed on the surface of the CNTs. Moreover, changing the thickness of the layer would also lead to the same response for each possibility. The oxidation of 4-hexylresorcinol was studied as a paradigm case with the modified electrode voltammetry showing the predicted but ambiguous response. Single-entity electrochemistry showed that the current–voltage response, notably the quasi-limiting current for the oxidation observed for single CNTs, was consistent with diffusion to the cylindrical CNT model, indicating that the ensemble voltammetry should be interpreted as resulting from the oxidation of solution phase analyte under thin-layer conditions. No evidence for adsorption effects was found.

Last, we present a *caveat*: having advocated the benefits of single-entity electrochemistry over ensemble measurements, we stress that all electrochemical measurements on nanomaterials reflect the composition and hence purity of the materials themselves. This is especially true of electrochemical investigations of ensembles since voltammetric responses of any kind are dominated in a potential sweep experiment by the most active sites within the material studied. The presence of just a small amount of especially active materials will lead to the discharge of the analyte under study at the potentials dictated by the unusually active material, so that when the potential scan reaches the potential required for the discharge on the dominant component there will be substantial depletion of the active material and the former response will mask the latter. Note that since diffusional depletion occurs over distances of tens to hundreds of micrometers within just a few seconds the overlap of the diffusional fields of trace impurities/active sites separated by these distances will be enough to ensure a diffusional response almost indistinguishable from that which would be seen if the entire surface was active at the lower potential. Figure 12 schematically illustrates the situation.

**Figure 12:**
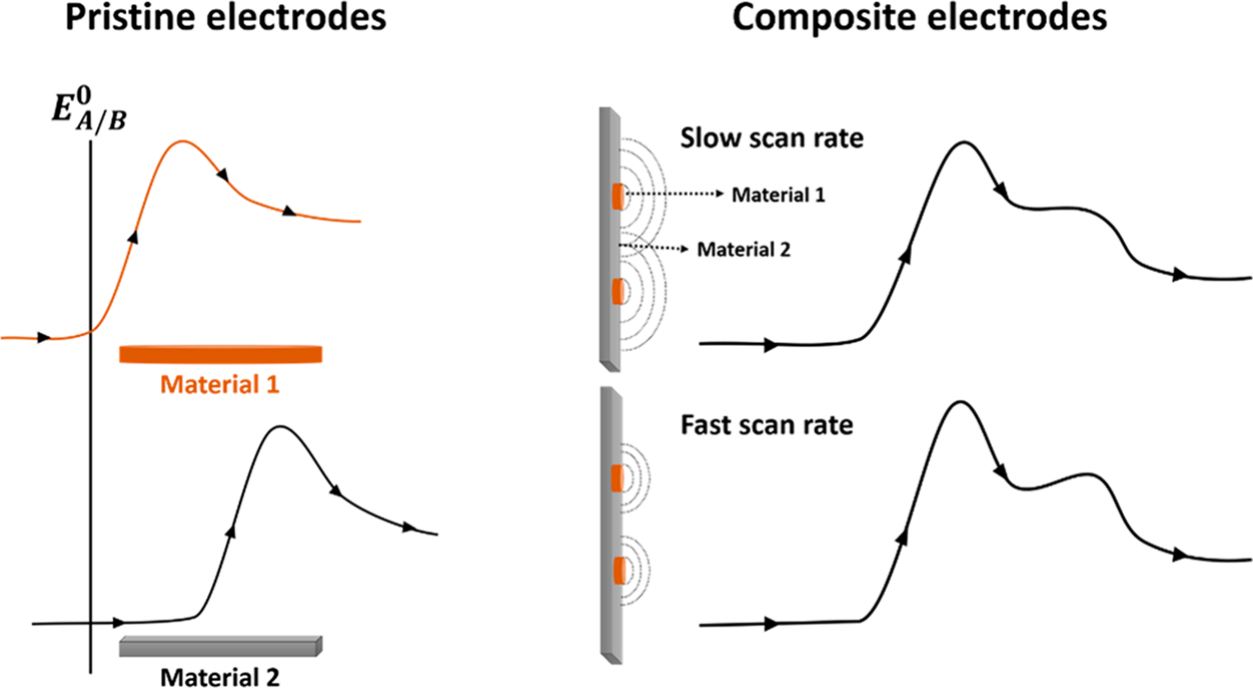
Comparative schematic illustrations of the voltammetric responses at pristine electrodes and at composites containing a small amount of a more active electrode material. Note the peak potential of the more electrocatalytic material is closer to the formal potential *E*_A/B_^0^.

CNTs often contain metal, metal oxide, and other impurities, for example arising from their synthesis where catalysts are often employed. It is important to distinguish between authentic CNT catalysis and that arising from these impurities when conducting voltammetric investigations of CNT electrocatalysis as noted first in 2006^56^ for the oxidation of epinephrine where trace iron oxide was deemed to be the actual catalytic material, and subsequently reported for diverse substrates, including halothane,^57^ hydrogen peroxide,^58^ and glucose,^59^ among others. Note that the influence of the trace impurities if they are more active than the dominant component of the nanocomposite depends not only on the impurity levels but also on the prevailing voltammetric time scale and the average distance between the impurities on the electrode surface. If the catalytic centers sustain a current at a lower potential than at the CNT, then if the associated diffusional fields expand to overlap before a voltage scan reaches the potential required for the electrolysis to take place on the CNT, a full voltammetric signal will be seen at the lower potential with only a negligible current flowing at the higher potential since the electroactive species is already depleted when the voltage scan reaches that potentials, as depicted in Figure 12. The latter implies that, for a given amount (mass %) of impurities, the extent to which it is physically dispersed is also crucial so that estimates of the ppm levels required to induce changed behavior^60^ need to be caveated accordingly. Note that purification of the CNTs, for example via acid washing, does not necessarily remove enough of the non-CNT material to be useful;^61^ rather, the use of metal catalyst-free CNTs is desirable.^62^ Similar considerations apply to other nanomaterials, notably graphene as elegantly demonstrated by Pumera and colleagues.^63,64^

To conclude, voltammetry at nanocomposites is complex and requires careful analysis. The above has summarized some of the pitfalls which despite being recognized for some time continue to reappear in the literature, especially in materials-focused publications. Recently, however, the appearance of single-entity electrochemistry offers an alternative approach for the more rigorous evaluation of electrocatalysts.

## Supporting Information Available

Examples of CNTs used in electroanalysis, energy applications, and electrocatalysis (Tables S1, S2, and S3, respectively); schematic showing the drop-casting technique (Figure S1) (PDF)
