# Supplementary material for: Voltammetry of Carbon Nanotubes and the Limitations of Particle-Modified Electrodes: Are Carbon Nanotubes Electrocatalytic?
Source: J Phys Chem Lett. 2022 Sep 12;13(37):8699–710. doi: 10.1021/acs.jpclett.2c02464 (PMC9511562; doi:10.1021/acs.jpclett.2c02464)
Supplement: Supplementary file 1 — jz2c02464_si_001.pdf [file jz2c02464_si_001.pdf]

## Supporting Information

# Voltammetry of Carbon Nanotubes and the Limitations of Particle Modified Electrodes: Are Carbon Nanotubes Electrocatalytic?

Archana Kaliyaraj Selva Kumar, Yuanyuan Lu, Richard G Compton*

*Department of Chemistry, Physical and Theoretical Chemistry Laboratory, Oxford University, South Parks Road, Oxford OX1 3QZ, Great Britain*

* Corresponding author.
E-mail: richard.compton@chem.ox.ac.uk (Richard Compton)

**Contents**

**Section 1:** Examples of CNT modified electrodes in electroanalysis

**Section 2:** Examples of CNT composite electrodes in batteries and fuel cells

**Section 3:** Schematic representation of the drop-casting technique

**Section 4:** Examples of CNT modified electrodes in electrocatalysis

## Section 1

**Table S1:** Examples of CNT modified electrodes in electroanalysis

| CNTs modified electrode | Application | Technique | Reference |
|---|---|---|---|
| Multiwalled carbon nanotubes (MWCNTs) modified glassy carbon (GC) electrodes | Detection of 2,4,6-trinitrotoluene | ASV | [1] |
| GC modified with MWCNTs and ionic liquids | Detection of ciprofibrate | DPV | [2] |
| MWCNTs modified screen printed electrodes | Detection of hesperidin | ASV | [3] |
| MWCNTs modified GC electrode | Detection of hydrocholothiazide (HCT) and triamterene (TRT) | stripping voltammetry | [4] |
| Au nanoparticles deposited CNTs modified GC electrode | Determination of As (III) | ASV | [5] |
| Functionalised MWCNTs modified GC electrode | Determination of paraquat | SWASV | [6] |
| CNTs on Teflon | Adrenaline sensing | DPV | [7] |
| CNTs on GC | $Cd^{2+}$ and $Pb^{2+}$ | SWASV | [8] |
| CNTs modified screen printed electrode | Vortioxetine hydrobromide | LSV | [9] |

| | | | |
|---|---|---|---|
| SWCNTs modified GC electrode | Dopamine, uric acid and ascorbic acid | SWASV | 10 |
| CNT – screen printed electrode | Organophosphorus pesticides | Amperometry | 11 |

MWCNTs, multiwalled carbon nanotubes; CNTs, carbon nanotubes; SWCNTs, single walled carbon nanotubes; GC, glassy carbon electrode; DPV, differential pulse voltammetry; ASV, adsorptive stripping voltammetry; SWASV, square-wave anodic stripping voltammetry; LSV. Linear sweep voltammetry

## Section 2

**Table S2** Examples of CNT composite electrodes in batteries and fuel cells

| CNTs electrode composite | Application | Reference |
|---|---|---|
| MWCNTs/tin oxide ($SnO_2$) nanocomposite | Anode in microbial fuel cell | [12] |
| Electrodeposited Pt-Ru and Pt-Ru-Ni nanoclusters on MWCNTs. | Electrocatalyst for ORR and MOR | [13] |
| Pd deposited MWCNTs | Anode in direct formic acid fuel cell | [14] |
| Graphene oxide/MWCNTs nanocomposite | Electrocatalyst for vanadium redox flow battery | [15] |
| $RuO_2$ modified CNTs | Cathode in Li-$O_2$ wearable battery | [16] |
| Nitrogen-doped CNTs | Cathode for lithium-air batteries | [17] |
| SWCNT doped with nitrogen and phosphorous. | Bifunctional oxygen electrocatalyst | [18] |
| Carbon nanofibers | Anode for microbial fuel cells | [19] |
| CNTs modified cathode | Li-S battery | [20] |
| SWCNTs with Si | Li-ion battery | [21] |

MWCNTs, multiwalled carbon nanotubes; CNTs, carbon nanotubes; SWCNTs, single walled carbon nanotubes; ORR, oxygen reduction reaction; MOR, methanol oxidation reaction.

**Section 3:** Schematic representation of the drop-casting technique

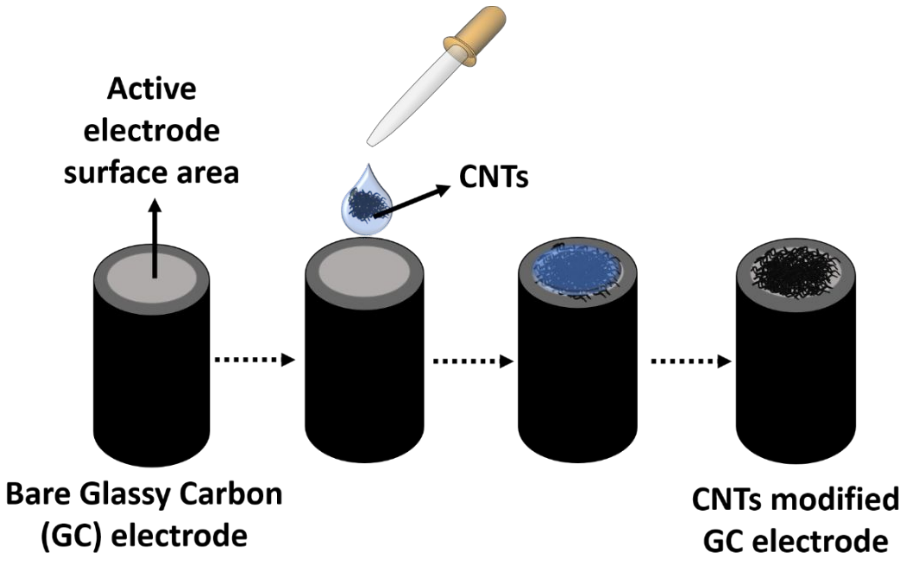


**Figure S1** A schematic representation of the drop-casting technique

## Section 4

**Table S3** Examples of CNT modified electrodes in electrocatalysis

| Electrode | Reaction or species studied | Electrocatalytic w.r.t | Reference |
|---|---|---|---|
| MWCNTs modified GC | $H_2O_2$ | GC | [22] |
| OCNTs modified GC | NADH oxidation | GC | [23] |
| MWCNT and SWCNT on GC | Catechol | GC | [24] |
| MWCNT and SWCNT on GC | ORR | GC | [25] |
| MWCNTs modified GC | Paracetamol | GC | [26] |
| SWCNTs on GC | Norepinephrine | GC | [27] |
| MWCNTs on GC | Nitric oxide | GC | [28] |
| MWCNTs on GC | $H_2O_2$ | GC | [29] |
| MWCNTs on GC | Cysteine | GC | [30] |
| MWCNTs on GC | Hydrazine | GC | [31] |
| MWCNTs on GC | Vitamin D3 oxidation | GC | [32] |
| MWCNTs on GC | Dopamine | GC | [33] |
| MWCNTs on GC | Hydroquinone and Catechol | GC | [34] |
| MWCNTs on GC | Choline | GC | [35] |
| MWCNTs on GC | AA and UA | GC | [36] |
| MWCNTs on GC | Glucose oxidase | GC | [37] |
| MWCNTs on CPE | AA, AC | CPE | [38] |
| MWCNTs on GC | $VO_2^+/VO^{2+}$ | GC | [39] |
| SWCNTs on CC | APAP, AA | CC | [40] |

| | | | |
|---|---|---|---|
| HCNTs on GC | AA, DA, and UA | GC | 41 |
| SWCNTs on GC | $VO_2^+/VO^{2+}$ and $V^{3+}/V^{2+}$ | GC | 42 |
| NCNTs on GC | ORR | GC | 43 |
| NCNTs on GC | ORR | GC | 44 |
| B-MWCNTs on GC | ORR/OER | GC | 45 |
| CNCNT on Ni electrode | ORR/OER | Ni | 46 |
| MWCNTs on GC | HER | GC | 47 |
| echo-MWCNTs on GC | OER | GC | 48 |
| NCNTs on GDE | Carbon dioxide | GDE | 49 |
| MWCNTs on GC | Cholecalciferol | GC | 50 |
| O-CNT on CP | Nitrogen | CP | 51 |

MWCNTs, multiwalled carbon nanotubes; OCNTs, ordered carbon nanotubes; SWCNTs, single walled carbon nanotubes; GC, glassy carbon electrode; NADH, Nicotinamide adenine dinucleotide; ORR, oxygen reduction reaction; APAP, acetaminophen, AA, ascorbic acid; CC, carbon–ceramic electrode; HCNTs, helical carbon nanotubes; UA, uric acid, DA, dopamine; NCNTs, N-doped carbon nanotubes; CPE, carbon paste electrode; AC, acetaminophen; B-MWCNTs, boron doped multi-walled carbon nanotubes; OER, oxygen evolution reaction; CNCNT, cobalt-embedded nitrogen doped carbon nanotubes; HER, hydrogen evolution reaction; echo-MWCNTs, surface oxidized multiwalled carbon nanotubes after hydrothermal treatment and electrochemical activation; GDE, gas diffusion electrode; O-CNT, oxidized carbon nanotube; CP, carbon paper.

## References


1. Wang, J.; Hocevar, S. B.; Ogorevc, B., Carbon nanotube-modified glassy carbon electrode for adsorptive stripping voltammetric detection of ultratrace levels of 2,4,6-trinitrotoluene. *Electrochemistry Communications* **2004,** *6* (2), 176-179.
2. Vicentini, F. C.; Ravanini, A. E.; Silva, T. A.; Janegitz, B. C.; Zucolotto, V.; Fatibello, O., A novel architecture based upon multi-walled carbon nanotubes and ionic liquid to improve the electroanalytical detection of ciprofibrate. *Analyst* **2014,** *139* (16), 3961-3967.
3. Sims, M. J.; Li, Q.; Kachoosangi, R. T.; Wildgoose, G. G.; Compton, R. G., Using multiwalled carbon nanotube modified electrodes for the adsorptive striping voltammetric determination of hesperidin. *Electrochim Acta* **2009,** *54* (22), 5030-5034.
4. Hudari, F. F.; Souza, J. C.; Zanoni, M. V. B., Adsorptive stripping voltammetry for simultaneous determination of hydrochlorothiazide and triamterene in hemodialysis samples using a multi-walled carbon nanotube-modified glassy carbon electrode. *Talanta* **2018,** *179*, 652-657.
5. Xiao, L.; Wildgoose, G. G.; Compton, R. G., Sensitive electrochemical detection of Arsenic (III) using gold nanoparticle modified carbon nanotubes via anodic stripping voltammetry. *Anal Chem Acta* **2008,** *620* (1-2), 44-49.
6. Garcia, L. L. C.; Figueiredo, L. C. S.; Oliveira, G. G.; Fatibello, O.; Banks, C. E., Square-wave voltammetric determination of paraquat using a glassy carbon electrode modified with multiwalled carbon nanotubes within a dihexadecylhydrogenphosphate (DHP) film. *Sensor Actuat B-Chem* **2013,** *181*, 306-311.
7. Charithra, M. M.; Manjunatha, J. G., Electrochemical sensing of adrenaline using surface modified carbon nanotube paste electrode. *Mater Chem Phys* **2021,** *262*.
8. Lu, Z. W.; Zhao, W. Y.; Wu, L.; He, J.; Dai, W. L.; Zhou, C. L.; Du, H. J.; Ye, J. S., Tunable electrochemical of electrosynthesized layer-by-layer multilayer films based on multi-walled carbon nanotubes and metal-organic framework as high-performance electrochemical sensor for simultaneous determination cadmium and lead. *Sensor Actuat B-Chem* **2021,** *326*.
9. El Henawee, M.; Saleh, H.; Attia, A. K.; Hussien, E. M.; Derar, A. R., Carbon nanotubes bulk modified printed electrochemical sensor for green determination of vortioxetine hydrobromide by linear sweep voltammetry. *Measurement* **2021,** *177*.
10. Yang, Y.; Li, M. X.; Zhu, Z. W., A novel electrochemical sensor based on carbon nanotubes array for selective detection of dopamine or uric acid. *Talanta* **2019,** *201*, 295-300.
11. Lin, Y. H.; Lu, F.; Wang, J., Disposable carbon nanotube modified screen-printed biosensor for amperometric detection of organophosphorus pesticides and nerve agents. *Electroanal* **2004,** *16* (1-2), 145-149.
12. Mehdinia, A.; Ziaei, E.; Jabbari, A., Multi-walled carbon nanotube/$SnO_2$ nanocomposite: a novel anode material for microbial fuel cells. *Electrochim Acta* **2014,** *130*, 512-518.
13. Zhao, Y.; Fan, L. Z.; Ren, J. L.; Hong, B., Electrodeposition of Pt-Ru and Pt-Ru-Ni nanoclusters on multi-walled carbon nanotubes for direct methanol fuel cell. *Int J Hydrogen Energ* **2014,** *39* (9), 4544-4557.
14. Mazurkiewicz-Pawlicka, M.; Malolepszy, A.; Mikolajczuk-Zychora, A.; Mierzwa, B.; Borodzinski, A.; Stobinski, L., A simple method for enhancing the catalytic activity of Pd deposited on carbon nanotubes used in direct formic acid fuel cells. *Appl Surf Sci* **2019,** *476*, 806-814.
15. Han, P. X.; Yue, Y. H.; Liu, Z. H.; Xu, W.; Zhang, L. X.; Xu, H. X.; Dong, S. M.; Cui, G. L., Graphene oxide nanosheets/multi-walled carbon nanotubes hybrid as an excellent electrocatalytic material towards $VO_2^+$/$VO^{2+}$ redox couples for vanadium redox flow batteries. *Energ Environ Sci* **2011,** *4* (11), 4710-4717.
16. Liu, T.; Xu, J. J.; Liu, Q. C.; Chang, Z. W.; Yin, Y. B.; Yang, X. Y.; Zhang, X. B., Ultrathin, lightweight, and wearable Li-$O_2$ battery with high robustness and gravimetric/volumetric energy density. *Small* **2017,** *13* (6).

17. Li, Y. L.; Wang, J. J.; Li, X. F.; Liu, J.; Geng, D. S.; Yang, J. L.; Li, R. Y.; Sun, X. L., Nitrogen-doped carbon nanotubes as cathode for lithium-air batteries. *Electrochemistry Communications* **2011,** *13* (7), 668-672.
18. Li, J. C.; Hou, P. X.; Cheng, M.; Liu, C.; Cheng, H. M.; Shao, M. H., Carbon nanotube encapsulated in nitrogen and phosphorus co-doped carbon as a bifunctional electrocatalyst for oxygen reduction and evolution reactions. *Carbon* **2018,** *139*, 156-163.
19. Cai, T.; Huang, M. H.; Huang, Y. X.; Zheng, W., Enhanced performance of microbial fuel cells by electrospinning carbon nanofibers hybrid carbon nanotubes composite anode. *Int J Hydrogen Energ* **2019,** *44* (5), 3088-3098.
20. Huang, J. Q.; Chong, W. G.; Zheng, Q. B.; Xu, Z. L.; Cui, J.; Yao, S. S.; Wang, C. W.; Kim, J. K., Understanding the roles of activated porous carbon nanotubes as sulfur support and separator coating for lithium-sulfur batteries. *Electrochim Acta* **2018,** *268*, 1-9.
21. Ikonen, T.; Kalidas, N.; Lahtinen, K.; Isoniemi, T.; Toppari, J. J.; Vazquez, E.; Herrero-Chamorro, M. A.; Fierro, J. L. G.; Kallio, T.; Lehto, V. P., Conjugation with carbon nanotubes improves the performance of mesoporous silicon as Li-ion battery anode. *Sci Rep,* **2020,** *10* (1).
22. Zhao, G. C.; Yin, Z. Z.; Zhang, L.; Wei, X. W., Direct electrochemistry of cytochrome c on a multi-walled carbon nanotubes modified electrode and its electrocatalytic activity for the reduction of H2O2. *Electrochemistry Communications* **2005,** *7* (3), 256-260.
23. Chen, J.; Bao, J. C.; Cai, C. X.; Lu, T. H., Electrocatalytic oxidation of NADH at an ordered carbon nanotubes modified glassy carbon electrode. *Anal Chim Acta* **2004,** *516* (1-2), 29-34.
24. Xu, Z.; Chen, X.; Qu, X. H.; Dong, S. J., Electrocatalytic oxidation of catechol at multi-walled carbon nanotubes modified electrode. *Electroanal* **2004,** *16* (8), 684-687.
25. Kruusenberg, I.; Alexeyeva, N.; Tammeveski, K.; Kozlova, J.; Matisen, L.; Sammelselg, V.; Solla-Gullon, J.; Feliu, J. M., Effect of purification of carbon nanotubes on their electrocatalytic properties for oxygen reduction in acid solution. *Carbon* **2011,** *49* (12), 4031-4039.
26. Hou, X. L.; Shen, G. J.; Meng, L.; Zhu, L.; Guo, M., Multi-walled carbon nanotubes modified glass carbon electrode and its electrocatalytic activity towards oxidation of paracetamol. *Russ J Electrochem* **2011,** *47* (11), 1262-1267.
27. Wang, J. X.; Li, M. X.; Shi, Z. J.; Li, N. Q.; Gu, Z. N., Electrocatalytic oxidation of norepinephrine at a glassy carbon electrode modified with single wall carbon nanotubes. *Electroanal* **2002,** *14* (3), 225-230.
28. Wu, F. H.; Zhao, G. C.; Wei, X. W., Electrocatalytic oxidation of nitric oxide at multi-walled carbon nanotubes modified electrode. *Electrochemistry Communications* **2002,** *4* (9), 690-694.
29. Wang, J.; Musameh, M., Carbon nanotube/teflon composite electrochemical sensors and biosensors. *Analytical Chemistry* **2003,** *75* (9), 2075-2079.
30. Chen, X.; Yang, Y.; Ding, M. Y., Electrocatalytic oxidation and sensitive detection of cysteine at layer-by-layer assembled carbon nanotube-modified electrode. *Anal Chim Acta* **2006,** *557* (1-2), 52-56.
31. Kamyabi, M. A.; Narimani, O.; Monfared, H. H., Electrocatalytic oxidation of hydrazine using glassy carbon electrode modified with carbon nanotube and terpyridine manganese(II) complex. *J Electroanal Chem* **2010,** *644* (1), 67-73.
32. Ramaprabhu, S.; Fathima, T. K. S., Evaluating the origin of the electrocatalytic activity of multiwalled carbon nanotubes towards Vitamin $D_3$ oxidation. *J Electroanal Chem* **2022,** *911*.
33. Yang, H. C.; Zhou, C. X.; An, J. J.; Yang, L. F.; Yang, Y. Y.; Liu, X. L., Ultra-fast synthesis of iron decorated multiwalled carbon nanotube composite materials: A sensitive electrochemical sensor for determining dopamine. *J Alloy Compd* **2022,** *897*.
34. Yang, M.; Guo, H.; Sun, L.; Wu, N.; Wang, M. Y.; Yang, F.; Zhang, T. T.; Zhang, J. Y.; Pan, Z. L.; Yang, W., Simultaneous electrochemical detection of hydroquinone and catechol using MWCNT-COOH/CTF-1 composite modified electrode. *Colloid Surface A* **2021,** *625*.
35. Uwaya, G. E.; Fayemi, O. E., Electrochemical detection of choline at f-MWCNT/$Fe_3O_4$ nanocomposite modified glassy carbon electrode. *Mater Res Express* **2021,** *8* (5).

36. Abellan-Llobregat, A.; Vidal, L.; Rodriguez-Amaro, R.; Canals, A.; Morallon, E., Evaluation of herringbone carbon nanotubes-modified electrodes for the simultaneous determination of ascorbic acid and uric acid. *Electrochim Acta* **2018,** *285*, 284-291.
37. Liu, Y. X.; Zhang, J.; Cheng, Y.; Jiang, S. P., Effect of carbon nanotubes on direct electron transfer and electrocatalytic activity of immobilized glucose oxidase. *ACS Omega* **2018,** *3* (1), 667-676.
38. Shahrokhian, S.; Asadian, E., Simultaneous voltammetric determination of ascorbic acid, acetaminophen and isoniazid using thionine immobilized multi-walled carbon nanotube modified carbon paste electrode. *Electrochimica Acta* **2010,** *55* (3), 666-672.
39. Li, W.; Liu, J.; Yan, C., Multi-walled carbon nanotubes used as an electrode reaction catalyst for VO2+/VO2+ for a vanadium redox flow battery. *Carbon* **2011,** *49* (11), 3463-3470.
40. Habibi, B.; Jahanbakhshi, M.; Pournaghi-Azar, M. H., Differential pulse voltammetric simultaneous determination of acetaminophen and ascorbic acid using single-walled carbon nanotube-modified carbon–ceramic electrode. *Analytical Biochemistry* **2011,** *411* (2), 167-175.
41. Cui, R.; Wang, X.; Zhang, G.; Wang, C., Simultaneous determination of dopamine, ascorbic acid, and uric acid using helical carbon nanotubes modified electrode. *Sensors and Actuators B: Chemical* **2012,** *161* (1), 1139-1143.
42. Li, W.; Liu, J.; Yan, C., The electrochemical catalytic activity of single-walled carbon nanotubes towards $VO_2^+/VO^{2+}$ and $V^{3+}/V^{2+}$ redox pairs for an all vanadium redox flow battery. *Electrochimica Acta* **2012,** *79*, 102-108.
43. Alexeyeva, N.; Shulga, E.; Kisand, V.; Kink, I.; Tammeveski, K., Electroreduction of oxygen on nitrogen-doped carbon nanotube modified glassy carbon electrodes in acid and alkaline solutions. *Journal of Electroanalytical Chemistry* **2010,** *648* (2), 169-175.
44. Deng, H.; Li, Q.; Liu, J.; Wang, F., Active sites for oxygen reduction reaction on nitrogen-doped carbon nanotubes derived from polyaniline. *Carbon* **2017,** *112*, 219-229.
45. Cheng, Y.; Tian, Y.; Fan, X.; Liu, J.; Yan, C., Boron doped multi-walled carbon nanotubes as catalysts for oxygen reduction reaction and oxygen evolution reactionin in alkaline media. *Electrochimica Acta* **2014,** *143*, 291-296.
46. Wang, Z.; Xiao, S.; Zhu, Z.; Long, X.; Zheng, X.; Lu, X.; Yang, S., Cobalt-embedded nitrogen doped carbon nanotubes: a bifunctional catalyst for oxygen electrode reactions in a wide ph range. *ACS Applied Materials & Interfaces* **2015,** *7* (7), 4048-4055.
47. Cui, W.; Liu, Q.; Cheng, N.; Asiri, A. M.; Sun, X., Activated carbon nanotubes: a highly-active metal-free electrocatalyst for hydrogen evolution reaction. *Chemical Communications* **2014,** *50* (66), 9340-9342.
48. Lu, X.; Yim, W.-L.; Suryanto, B. H. R.; Zhao, C., Electrocatalytic oxygen evolution at surface-oxidized multiwall carbon nanotubes. *J. Am Chem Soc* **2015,** *137* (8), 2901-2907.
49. Wu, J.; Yadav, R. M.; Liu, M.; Sharma, P. P.; Tiwary, C. S.; Ma, L.; Zou, X.; Zhou, X.-D.; Yakobson, B. I.; Lou, J.; Ajayan, P. M., Achieving highly efficient, selective, and stable $CO_2$ reduction on nitrogen-doped carbon nanotubes. *ACS Nano* **2015,** *9* (5), 5364-5371.

50. KAHYA, Ş. E.; CİTTAN, M.; ÇELİK, A., Electrochemical behavior of cholecalciferol on a multiwalled carbon nanotube modified glassy carbon electrode. *Cumhuriyet Science Journal* **2018,** *39* (4), 1081-1088.
51. Zhao, J.; Wang, B.; Zhou, Q.; Wang, H.; Li, X.; Chen, H.; Wei, Q.; Wu, D.; Luo, Y.; You, J., Efficient electrohydrogenation of $N_2$ to $NH_3$ by oxidized carbon nanotubes under ambient conditions. *Chemical Communications* **2019,** *55* (34), 4997-5000.
